# The DNA-damage signature in *Saccharomyces cerevisiae *is associated with single-strand breaks in DNA

**DOI:** 10.1186/1471-2164-7-313

**Published:** 2006-12-12

**Authors:** Rebecca C Fry, Michael S DeMott, Joseph P Cosgrove, Thomas J Begley, Leona D Samson, Peter C Dedon

**Affiliations:** 1 Department of Biological Engineering, Massachusetts Institute of Technology, NE47-277, 77 Massachusetts Avenue, Cambridge, MA 02139, USA; 2 Center for Environmental Health Sciences, Massachusetts Institute of Technology, 56-235, 77 Massachusetts Avenue, Cambridge, MA 02139, USA; 3 Novartis Institute for Biomedical Research, 250 Massachusetts Ave., 3C-141, Cambridge, MA 02139, USA; 4 University of Albany, Gen*NY*Sis Center, Room 211, 1 Discovery Drive, Rensselear, NY 12144-3456, USA

## Abstract

**Background:**

Upon exposure to agents that damage DNA, *Saccharomyces cerevisiae *undergo widespread reprogramming of gene expression. Such a vast response may be due not only to damage to DNA but also damage to proteins, RNA, and lipids. Here the transcriptional response of *S. cerevisiae *specifically induced by DNA damage was discerned by exposing *S. cerevisiae *to a panel of three "radiomimetic" enediyne antibiotics (calicheamicin γ_1_^I^, esperamicin A1 and neocarzinostatin) that bind specifically to DNA and generate varying proportions of single- and double-strand DNA breaks. The genome-wide responses were compared to those induced by the non-selective oxidant γ-radiation.

**Results:**

Given well-controlled exposures that resulted in similar and minimal cell death (~20–25%) across all conditions, the extent of gene expression modulation was markedly different depending on treatment with the enediynes or γ-radiation. Exposure to γ-radiation resulted in more extensive transcriptional changes classified both by the number of genes modulated and the magnitude of change. Common biological responses were identified between the enediynes and γ-radiation, with the induction of DNA repair and stress response genes, and the repression of ribosomal biogenesis genes. Despite these common responses, a fraction of the response induced by gamma radiation was repressed by the enediynes and *vise versa*, suggesting that the enediyne response is not entirely "radiomimetic." Regression analysis identified 55 transcripts with gene expression induction associated both with double- or single-strand break formation. The *S. cerevisiae *"DNA damage signature" genes as defined by Gasch *et al*. [1] were enriched among regulated transcripts associated with single-strand breaks, while genes involved in cell cycle regulation were associated with double-strand breaks.

**Conclusion:**

Dissection of the transcriptional response in yeast that is specifically signaled by DNA strand breaks has identified that single-strand breaks provide the signal for activation of transcripts encoding proteins involved in the DNA damage signature in *S. cerevisiae*, and double-strand breaks signal changes in cell cycle regulation genes.

## Background

Exposure to DNA damaging agents can cause mutation and cell death and may ultimately lead to disease. Protection from this damage is provided by a host of DNA repair and cell cycle checkpoint proteins that collectively represent numerous pathways to help in the recovery response [2]. In humans, there are approximately 150 DNA repair and cell cycle checkpoint proteins that serve to ensure the repair of damage caused to DNA [3,4] and most of these have functional homologues in *S. cerevisiae*. Recently, studies have shown that proteins with additional cellular functions beyond DNA repair and cell cycle regulation may ameliorate the toxic effects of agents that cause DNA damage [5-8].

Genome-wide phenotyping studies to identify genes involved in cellular recovery after exposure to DNA alkylating agents, such as methyl methane sulfonate (MMS), indicate that a vast array of cellular processes are required for the recovery of *S. cerevisiae*. Transcriptional profiling demonstrated that up to 30% of the *S. cerevisiae *~6000 genes respond upon exposure to MMS in a time-, agent- and dose-dependent manner [9,10]. Given this vast transcriptional response to damaging agents like MMS and the knowledge that, in addition to DNA, these agents can also damage proteins, RNA and lipids, we set out to identify the transcriptional response specifically caused by damage to DNA.

**Figure 1:**
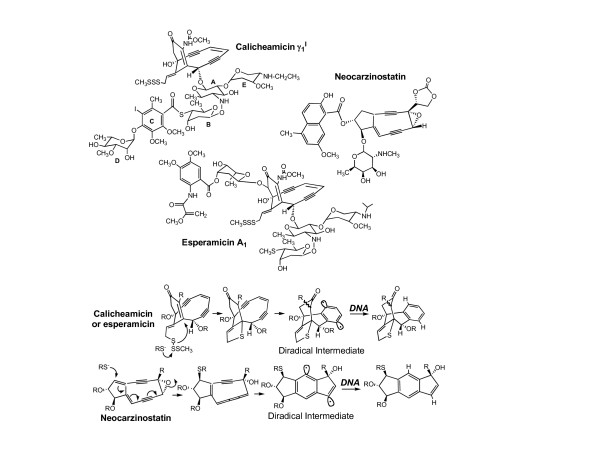
Chemical structures and relative ratios of double-strand to single-strand lesions generated. Three enediynes were used for treatment in *S. cerevisiae *including calicheamicin γ_1_^I^, esperamicin A1 and neocarzinostatin. Each of these results in varying ratios of double-strand (DS) to single-strand (SS) lesions in DNA as outlined in Table 1.

Here we compared the responses of *S. cerevisiae *upon exposure to γ-radiation, a non-selective oxidant that attacks DNA (base and sugar), lipids, carbohydrates, proteins and small metabolites in cells, with the response of *S. cerevisiae *to a panel of enediyne antibiotics that are known to damage DNA and not other cellular molecules (calicheamicin γ_1_^I^, esperamicin A1 and neocarzinostatin; structures shown in Figure 1) [11,12]. The enediyne family is a structurally diverse group of DNA-cleaving molecules that undergo reductive activation, presumably by glutathione *in vivo*, to form a diradical intermediate that binds with high affinity (10^9 ^M^-1^) in the minor groove of DNA and abstract hydrogen atoms from deoxyribose. This "radiomimetic" damage leads to the formation of well-defined proportions of single- and double-stranded DNA lesions unique to each type of enediyne [11,12] the proportions of which are noted in Table 1. With no damage to DNA bases, the enediynes specifically oxidize the deoxyribose moiety to produce either direct strand breaks with various sugar residues or phosphate groups attached to the 3'- or 5'-ends of the breaks, or various unstable oxidized abasic sites [11].

Expression profiling experiments have been performed using some of these agents independently [13,14]. However, there are no studies that directly compare the responses of these enediynes to each other and to that of γ-radiation under identical conditions. We establish four key findings: (i) under conditions of similar cell survival, exposure to non-selective γ-radiation results in more extensive reprogramming of *S. cerevisiae *transcription than does exposure to the DNA-selective enediynes; (ii) in response to DNA-strand breaks induced by both the non-selective and selective treatments, *S. cerevisiae *induces genes involved in DNA repair and the general stress response and represses genes encoding for ribosomal biogenesis; (iii) a considerable fraction of the response upon exposure to γ-radiation was not mimicked by treatment with the enediynes; and (iv) the "DNA damage signature" as described by Gasch *et al*. [1] in *S. cerevisiae *is associated with single-strand breaks in DNA. These results have implications for our understanding of the cellular responses arising from damage to specific components of the cell.

## Results and discussion

### Genome-wide responses of *S. cerevisiae *upon exposure to enediynes and γ-radiation are partially overlapping

**Figure 2:**
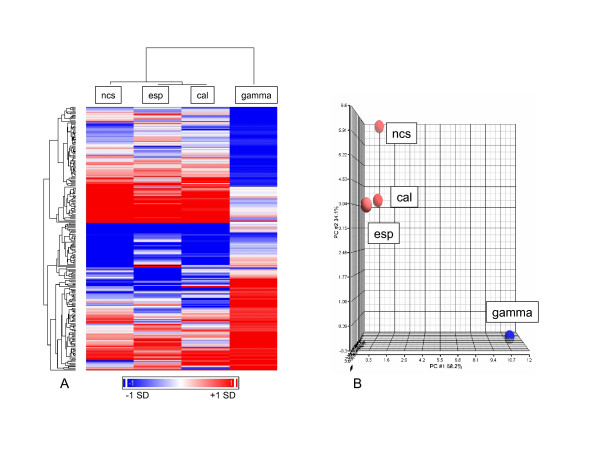
Hierarchical cluster and principal component analysis of damage-induced gene expression ratios. WT *S. cerevisiae *was exposed to damage induced by enediynes: calicheamicin γ_1_^I ^(cal), esperamicin A1 (esp) and neocarzinostatin (ncs) or γ-radiation (gamma). Significantly modulated transcripts in any one experiment (see Methods) were identified resulting in 225 ORFs and clustered hierarchically (A) or analyzed for principal components (B).

The expression patterns of yeast genes were monitored in *S. cerevisiae *exposed to the enediynes calicheamicin γ_1_^I^, esperamicin A1 and neocarzinostatin, and to γ-radiation. Care was taken in these studies to control the conditions of the treatments, including cytotoxicity (doses producing ~20–25% lethality; [see Additional files 3 and 4]), exposure time (15 min for all chemicals; for γ-radiation, cells were incubated for 13 min at ambient temperature following the ~2 min irradiation), and exposure temperature (ambient temperature for all exposures). The reproducibility of the dose-lethality relationships of the agents is indicated by the highly similar concentrations of calicheamicin and neocarzinostatin producing 20–25% lethality in the DBY747 strain employed for these studies and in another strain, BY4741: 1–1.4 nM *vs*. 0.8–1 nM for calicheamicin, and 3.3–4.2 nM *vs*. 2–3 nM for neocarzinostatin, respectively [see Additional files 3 and 4]. Statistically significant transcriptional changes were determined for treated cells *versus *untreated (water or methanol vehicle for irradiated and enediyne-treated cells, respectively) using local pooled error analysis with a correction for false discovery rate (Table 2). The stringent selection criteria for gene expression change (see Methods) identified changes in 225 ORFs (~4% of the genome) across all experiments. Hierarchical cluster analysis displays the similarities and differences between the expression profiles of the enediyne and γ-radiation treated *S. cerevisiae *(Figure 2A). The highest similarities in expression responses were seen for the three enediyne treatments, which showed more similarity to each other than to γ-radiation as visualized in the dendrogram in the hierarchical cluster (Figure 2A). The difference between the response of the enediynes and γ-radiation was also identified using principal component analysis (PCA) with the highest similarity found between the responses of the enediynes, which clustered separately from γ-radiation (Figure 2B). PCA showed that the largest percentage of variability within the dataset (58.2%) was attributable to treatment effect (Figure 2B). Although *S. cerevisiae *treated with the enediynes did show highly similar responses in genome-wide modulation relative to γ-radiation, there were minor differences within the responses induced by neocarzinostatin, calicheamicin and esperamicin. These differences influence the presence of the subdendrogram generated via hierarchical cluster analysis within the enediynes (Figure 2A), as well as the principal component analysis where neocarzinostatin response differs moderately from that of calicheamicin and esperamicin (Figure 2B). Although these minor differences exist within the response to enediyne treatment, the overall responses were highly similar to each other and strikingly different to that of γ-radiation.

### Common and unique responses of *S. cerevisiae *upon exposure to enediynes or γ-radiation

**Figure 3:**
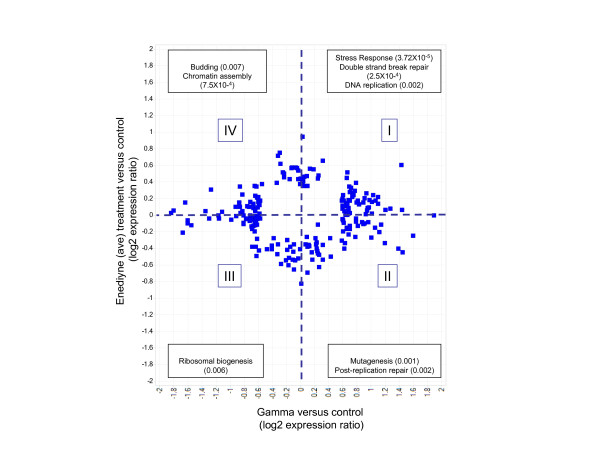
Scatter plot of relative transcriptional modulation in enediyne or γ-irradiated *S. cerevisiae*. The average expression ratio for enediynes was compared to that of γ-irradiated cells. Four primary sectors are identified (I-IV) demonstrating common or unique responses. Subsets of biological processes enriched (see Methods) are shown within each of the sectors [for complete list see Additional file 1].

As shown in Figure 3, comparisons of the gene expression responses of the collective enediynes to γ-radiation identify four primary sectors: (I) those that were similarly induced upon exposure to enediynes and γ-radiation; (II) those that were induced upon exposure to γ-radiation but repressed upon exposure to enediynes; (III) those that were similarly repressed upon exposure to enediynes and γ-radiation; and (IV) those that were repressed upon exposure to γ-radiation and induced by enediynes (Figure 3). Gene Ontology enrichment analysis identified biological processes significantly enriched within each of these four sectors (Figure 3 and [see Additional file 1]). In general, the absolute fold change of the enediyne treatment was lower than fold changes observed with γ-radiation. We hypothesize that this difference is based on the specificity of the damage generated by the enediynes *versus *the nonspecific nature of the cellular damage caused by exposure to γ-radiation.

Common responses were identified to treatment with both enediynes and γ-radiation representing 55% of the modulated transcripts (124 of 225 ORFs). Genes induced by both enediynes and γ-radiation (Sector I) were represented by 13 Gene Ontology categories, 7 of which are involved in DNA repair [see Additional file 1]. The most significant class enriched was that of Response to Stress (p < 3.7 × 10^-5^) and includes *MSN2, MSN4, DUN1*, and *RAD51*. Genes repressed by both enediynes and γ-radiation (Sector III) were represented by 31 Gene Ontology categories. Among these 31 categories, significant enrichment was seen in the Ribosomal Large Subunit Biogenesis (p < 0.006) [see Additional file 1]. Interestingly, both the induction of stress response genes and repression of ribosomal protein synthesis genes are two of the hallmarks of the Environmental Stress Response (ESR) in *S. cerevisiae*. The ESR was identified by Brown and colleagues where a commonly responding set of genes (~900) was modulated upon exposure to a wide variety of environmental stressors including temperature shock, hydrogen peroxide and hyper- and hypo-osmotic shock [15].

Despite the common response to treatment with enediynes and γ-radiation described above, a substantial fraction (45%) of the genomic response induced by γ-radiation was repressed by the enediynes and *vise versa *suggesting that much of the response to the enediynes is not "radiomimetic." Specifically 101 transcripts of the total 225 modulated transcripts demonstrated opposite directionality between γ-radiation and the enediynes. Genes that were induced by γ-radiation but repressed by enediynes (Sector II) were enriched for 19 Gene Ontology categories [see Additional file 1]. The most significantly enriched class was Mutagenesis (p < 2 × 10^-6^) and includes *POL30 *and *CDC8*. The final class included transcripts repressed upon exposure to γ-radiation and induced by enediynes (Sector IV). A significantly enriched Gene Ontology class includes Chromatin Assembly/Disassembly (p < 0.0007) including *HTB2, HHT1*, and *HHT2 *[see Additional file 1].

### Regression analysis identified gene induction that associates with double-strand or single-strand damage

**Figure 4:**
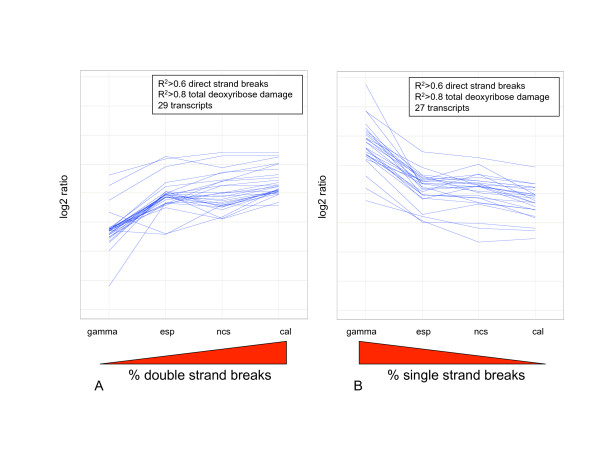
Trend line graph of ORFS with transcriptional modulation associated with single-strand or double-strand DNA breaks. ORFs with associated transcriptional modulation in response to treatment with enediynes or γ-radiation were identified using regression analysis (see Methods). A) ORFs with modulation associated with double-strand breaks and B) ORFs with modulation associated with single-strand breaks [for complete list of ORFs see Additional file 2].

Using the various proportions of single- and double-strand deoxyribose oxidation data shown in Table 1, we performed regression analysis of gene expression responses to treatment with each of the enediynes and γ-radiation. The results identified 55 ORFs with transcriptional induction that associates either with double-strand or single-strand damage (Figure 4A and 4B, respectively). As shown in Table 3, positive association was identified with 29 ORFs demonstrating elevated expression in response to double-strand lesions and 27 ORFS with elevated expression in response to single-strand lesions (Table 3). While these correlations are significant (r^2 ^> 0.6) for direct strand breaks, the correlation increases substantially when total deoxyribose oxidation is considered (r^2 ^> 0.8; Table 3). The distinction between direct strand breaks and total deoxyribose oxidation involves the formation of oxidized abasic sites by γ-radiation and the enediynes. These lesions consist of the 2'-deoxyribonolactone and 2-deoxypentose-4-ulose derived from 1'- and 4'-oxidation of deoxyribose in DNA, respectively, and are present in more than 75% of calicheamicin- and neocarzinostatin-induced double-strand lesions DNA damage [12]. The abasic sites are not taken into account in most studies of "strand break" formation by oxidizing agents (*e.g*., see ref. [16]. Indeed, the 1:3 ratio of direct double- to single-strand breaks observed by Hammersten and coworkers for calicheamicin-induced damage in cells [16] is similar to the 1:1 to 1:2 ratio for direct breaks that they and others observed in plasmid DNA *in vitro *[12,16-18]. Hydrolysis of the abasic sites dramatically shifts the ratio of lesions toward double-strand breaks, as shown in Table 1. The abasic sites are DNA lesions that cause significant distortion of the DNA helix, so they may indeed be recognized by proteins involved in the detection of double-strand DNA lesions, as suggested by the stronger correlation observed for total deoxyribose oxidation than for direct strand breaks (Table 3), an hypothesis that warrants further study.

Interestingly, ORFs with regulatory association with single-strand breaks show an enrichment of gene ontology categories including Response to Stress (*HSP26, YCL033C, DUN1, RAD51, HYR1, ASF1, AHP1, CRS5*), and DNA replication (*POL1, RFA2*) [see Additional file 2]. Within this single-strand break associated gene set there is an enrichment of genes belonging to the "DNA damage signature" in *S. cerevisiae *including *DUN1*, *RAD51*, *RNR2*, and *YBR070C*. The DNA damage signature, identified by Brown and colleagues, includes nine genes: the DNA damage repair genes *RAD51 *and *RAD54*, ribonucleotide reductase subunits *RNR2 *and *RNR4*, DNA damage activated kinase *DUN1*, and uncharacterized genes *YER004W *and *YBR070C *[1]. The "DNA damage" signature was identified by comparing expression programs in *S. cerevisiae *that were modulated by a range of environmental stresses including heat shock, oxidative stress, reductive stress, osmotic shock and amino acid starvation to those elicited specifically by DNA damage induced by the alkylating agent methylmethane sulfonate (MMS) and ionizing radiation (γ-radiation) [1,15]. Although it is not surprising that here we identify the DNA damage signature gene set as associated with γ-radiation (as this gene set was characterized in response to γ-radiation), it is noteworthy that there is a specificity of the activation of the DNA damage signature gene set to single-strand damage.

**Figure 5:**
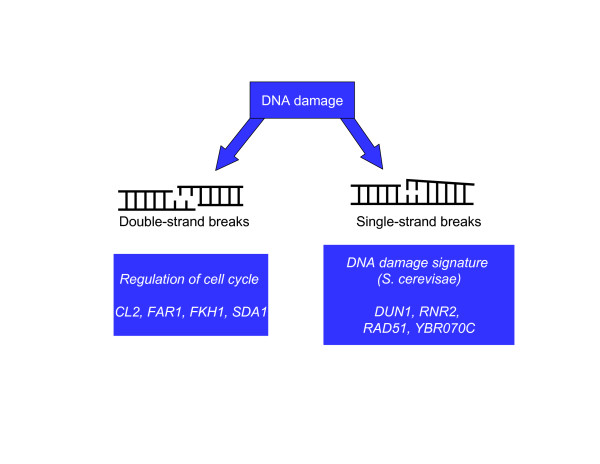
Model of specific responses in *S. cerevisiae *to double- or single-strand breaks.

The induction of the DNA damage signature gene set was muted in cells deficient for the ATR homolog, *mec1*, as well as cells deficient for *dun1*, which indicates these genes are downstream and targets for the Mec1 pathway [1]. In contrast to the single-strand break associated gene set, genes associated with double-strand damage are enriched for gene ontology categories that include cell cycle regulation [see Additional file 2]. Interestingly, no DNA repair or stress responses categories were significantly enriched within this gene set. These results suggest that there is differential genomic modulation in response to single-strand or double-strand DNA damage in *S. cerevisiae *(Figure 5).

### Comparisons with published studies of enediyne-induced changes in gene expression

Expression profiling experiments have been performed using calicheamicin [13] and neocarzinostatin [14]. Several major experimental differences make it impossible to directly compare the data generated here with those of the published studies. The calicheamicin studies performed by Schaus *et al*. employed the YPH500α strain of *S. cerevisiae *and significantly higher (8–80 nM) drug concentrations [13], while the neocarzinostatin studies of Watanabe *et al*. [14] utilized strain BY4741 and the holoantibiotic form of the drug (DNA-cleaving chromophore bound to 17,000 Da apo-protein; see ref. [11]). In addition to the absence of publicly available expression data sets for these studies, neither reported any measure of enediyne-induced cytotoxicity or other biological response that could be used to normalize the resulting transcriptional data.

## Conclusion

Genome-wide expression profiling in *S. cerevisiae *has resulted in the understanding that a robust response is mounted upon exposure to agents that damage DNA. Here we discern the components of the transcriptional response of *S. cerevisiae *that are specifically due to particular types of DNA damage, namely that of single- or double-strand DNA damage arising from deoxyribose oxidation. These studies were conducted with a panel of three DNA-selective enediyne antibiotics (calicheamicin γ_1_^I^, esperamicin A1 and neocarzinostatin) that produce different proportions of double- and single-strand deoxyribose damage in DNA, with genome-wide responses compared to those induced by the non-selective γ-radiation. We find at doses producing similar toxicity, exposure to non-selective γ-radiation results in more extensive reprogramming of the *S. cerevisiae *transcriptome than exposure to each of the three enediynes. The extensive response to γ-radiation may reflect the non-specific nature of the oxidative attack on DNA (base and sugar), lipids, carbohydrates, proteins and small metabolites in cells. A striking finding is that only a modest fraction of the response upon exposure to γ-radiation was mimicked by treatment with the enediynes. We find that, in response to DNA strand breaks induced by both the non-selective and selective agents, yeast induce genes involved in DNA repair and stress response and repress genes encoding for ribosomal biogenesis. We also identify that the DNA damage signature in *S. cerevisiae *is more closely associated with single-strand breaks in DNA, than with double-strand breaks.

## Methods

### Yeast strains, culture, and reagents

*Saccharomyces cerevisiae *strain DBY747 (*MAT*a, *his3*Δ1, *leu2*Δ112, *ura3*Δ52, *trp1*Δ289a gals, Can1, CUPr) was used in this study. Enediynes were used as methanolic stocks and obtained as follows: Calicheamicin was obtained from Wyeth Research (Cambridge, MA), neocarzinostatin was obtained from Kayaku Co. Ltd. (Tokyo, Japan; no longer available; currently produced by Sigma Chemical Company, St. Louis, MO), esperamicin A1 was obtained from Bristol Myers Squibb (Wallingford, CT; no longer available). The neocarzinostatin chromophore was isolated by methanol extraction and the concentration determined as described elsewhere [19]. Cells were grown and maintained in YPD (10 g yeast extract, 20 g peptone, 20 g dextrose, 20 g agar per liter). As shown in [Additional file 3], samples of the DBY747 WT strain were grown to mid-log phase and treated with doses of agents determined to produce ~20–25% lethality following a 15 min exposure: calicheamicin (1 nM), neocarzinostatin (4 nM), esperamicin A1 (3 nM), and γ-radiation (210 Gy delivered at 105 Gy/min in a ^60^Co source with 13 min post-irradiation incubation at ambient temperature). Similar studies were performed with the BY4741 strain, with similar results [see Additional file 4]. All exposures were performed at ambient temperature under identical conditions of light and temperature, and cells were snap frozen in liquid nitrogen immediately after the 15 min exposure period.

### RNA preparation/cRNA synthesis

Total RNA was extracted from pelleted cells using a hot phenol protocol [20] that has been used successfully in previous expression profiling studies [5,9,10], including one with calicheamicin [14]. AE buffered phenol (50 mM sodium acetate pH 5.3, 10 mM EDTA, pH 8.0) was utilized. RNA was precipitated from the aqueous phase of the phenol extraction. Labeled cRNA was generated as follows. Total RNA was converted into single-stranded cDNA using a modified oligo(dT) primer with a 5' T7 RNA polymerase promoter sequence and reverse transcriptase (SuperScript II RT, Gibco). Double-stranded cDNA was generated using DNA polymerase and DNA ligase (Invitrogen Life Technologies) and purified. Biotin-labeled cRNA was generated using *in vitro *transcription with T7 RNA polymerase (ENZO BioArray HighYield RNA transcript Labeling Kit, Affymetrix, CA). Reactions were carried out for 5 h at 37°C and cRNA was purified using RNeasy spin columns (Qiagen, CA). cRNA was quantified at UV_260 _and 15 μg of RNA was fragmented randomly using (200 mM Tris-Acetate, 500 mM potassium acetate, 150 mM magnesium acetate) at 94°C for 35 min.

### GeneChip^® ^hybridizations and image analysis

Hybridizations were performed as follows. Fragmented cRNA was hybridized to GeneChip^® ^(YG-S98, Affymetrix, CA) at a concentration of 0.05 μg/μl in 200 μl of Affymetrix buffer (100 mM MES, 1 M NaCl, 20 mM EDTA, 0.01% Tween 20) with GeneChip^® ^eukaryotic hybridization controls (GeneChip^® ^Eukaryotic Hybridization Controls Kit, Affymetrix, CA) in the presence of 0.1 mg/ml herring sperm DNA and 0.5 mg/ml acetylated BSA at 40°C for 16 h with constant rotation. Arrays were rinsed after hybridization with 200 μl of stringent wash buffer (100 mM MES, 0.1 M NaCl, 0.01% Tween 20) followed by a non-stringent wash (6XSSPE, 0.01% Tween 20). 20XSSPE had the following composition (3 M NaCl, 0.2 M NaH_2_PO_4_, 0.02 M EDTA). Staining was done with 2 μg/ml streptavidin-phycoerytherin and 1 mg/ml acetylated BSA in 6xSSPE-T. Arrays were scanned using a HP G2500A GeneArray scanner.

### Data pre-processing and differential gene testing

Untreated and treated samples were analyzed in biological triplicate on YG-S98 arrays. Quantile normalization was carried out using the Robust Multichip Average (RMA) algorithm [21]. Transcripts that were absent across all experiments were identified using Absent/Present calls (Microarray Suite 5.0) and filtered for transcripts that were not expressed in any experiment. Differential gene expression was calculated using the dual filtering criteria of ≥1.5 fold change and statistical significance determined through the Local Pooled Error test (LPE) [22] with an adjustment for false discovery rate calculation of p value of ≤0.10 [23]. All microarray data have been submitted to the Gene Expression Omnibus Database (Series, GSE5301).

### Gene Ontology Enrichment Analysis

Statistical evaluation of co-regulated groups of genes was carried out through Gene Ontology Enrichment Analysis within the Functional Specification Database (funspec.med.utoronto.ca) [24]. Co-regulated yeast ORFs were classified according to the Gene Ontology Biological Process and the hypergeometric distribution (p < 0.01) used to assess enrichment of a particular gene category. The hypergeometric distribution asses for each gene ontology category, the probability (p-value) of observing such an overlap by chance is calculated as:

P=1−∑i=0k−1(Ci)(G−Cn−i)(Gn)
 MathType@MTEF@5@5@+=feaafiart1ev1aaatCvAUfKttLearuWrP9MDH5MBPbIqV92AaeXatLxBI9gBaebbnrfifHhDYfgasaacH8akY=wiFfYdH8Gipec8Eeeu0xXdbba9frFj0=OqFfea0dXdd9vqai=hGuQ8kuc9pgc9s8qqaq=dirpe0xb9q8qiLsFr0=vr0=vr0dc8meaabaqaciaacaGaaeqabaqabeGadaaakeaacqWGqbaucqGH9aqpcqaIXaqmcqGHsisldaaeWbqaamaalaaabaWaaeWaaeaafaqabeGabaaabaGaem4qameabaGaemyAaKgaaaGaayjkaiaawMcaamaabmaabaqbaeqabiqaaaqaaiabdEeahjabgkHiTiabdoeadbqaaiabd6gaUjabgkHiTiabdMgaPbaaaiaawIcacaGLPaaaaeaadaqadaqaauaabeqaceaaaeaacqWGhbWraeaacqWGUbGBaaaacaGLOaGaayzkaaaaaaWcbaGaemyAaKMaeyypa0JaeGimaadabaGaem4AaSMaeyOeI0IaeGymaedaniabggHiLdaaaa@49FD@

where *G *is the size of the genome, *C *is the number of genes in the genome having that attribute, *n *is the size of the query cluster, of which k are known to possess the attribute.

### Regression analysis

To identify genes with expression associated with double- or single-strand damage, all modulated ORFs across experimental conditions (225 total) were analyzed for association with induced expression ratio relative to percent single or double-strand lesion formation. Linear regression was performed on expression ratios relative to percent of direct single-strand or double-strand breaks with R^2^>0.6 and percent of total deoxyribose oxidation events with R^2^>0.8 selected for associated gene sets (Figure 4).

## Authors' contributions

RF carried out the analysis of all genomics experiments and drafted the manuscript. MD drafted the manuscript and participated in the analysis of the genomics experiments. JC carried out the experimental treatments of *S. cerevisiae *and the hybridizations to GeneChips. TB assisted in the experimental treatments and gene expression profiling experiments. LS participated in the oversight of the study and helped to draft the manuscript. PD conceived of the study and participated in its design and coordination and helped to draft the manuscript. All authors read and approved the final manuscript.

## Supplementary Material

Additional File 1 Differential enrichment of GO categories identified in the comparison of yeast responses to enediynes and γ-radiation. The table lists the gene ontology categories identified as significantly enriched within the four sectors identified in the comparison of the enediynes response to that of γ-radiation. Click here for file

Additional File 2 GO categories identified as associated with single-strand or double-strand damage. The table is a list of the gene ontology categories enriched in the gene sets that were identified as associated with single-strand or double-strand damage. Click here for file

Additional File 3 Cytotoxicity dose-response curves for *S. cerevisiae *strain DBY747 treated with enediynes. The figure plots the cytotoxicity dose-response curve for exposure of strain DBY747 to enediynes. Click here for file

Additional File 4 Cytotoxicity dose-response curves for *S. cerevisiae *strain BY4741 treated with enediynes. The figure plots the cytotoxicity dose-response curve for exposure of strain BY4741 to enediynes. Click here for file

## Figures and Tables

**Table 1:** Proportions of Single- and Double-Strand Damage Produced by γ-Radiation and Enediynes

**Agent**	**Ratio of direct single- to double-strand breaks**	**Ratio of single- to double-strand deoxyribose oxidation events**
γ-Radiation	35–100:1^a^	35–100:1^a^
Esperamicin A1	24:1^b^	3.4:1^b^
Neocarzinostatin	6:1^c^	2.2:1^c^
Calicheamicin-γ_1_^I^	1:1^d^	1:24^d^

^a ^Reference [25, 26]

^b ^Reference [29]

^c ^Reference [30]

^d ^Reference [12, 31]

**Table 2:** Gene expression changes in response to enediyne or γ-radiation treatment^a^

	**# of unique ORFS with ≥ 1.5-fold change**^**b**^		
	**Increased Expression**	**Decreased Expression**	**Total**

**Calicheamicin γ**_**1**_^**I**^	4	3	7
**Esperamicin A1**	5	13	18
**Neocarzinostatin**	26	37	63
**γ-Radiation**	77	74	151

^a ^WT *S. cerevisiae *was exposed to enediynes or γ-radiation and their relative transcriptomes compared. Total numbers of unique ORFs are represented that showed significant modulation (see Methods).

^b ^Adjusted p-value ≤ 0.1

**Table 3:** Elevated expression of ORFs associated with single- and double-strand damage caused by exposure of *S. cerevisiae *to γ-radiation and enediynes^a^

**Elevated Expression of ORFS Associated with Single-Strand Damage**			**Elevated Expression of ORFS Associated with Double-Strand Damage**		
***ORF***	***Gene Name***	***Description***	***ORF***	***Gene Name***	***Description***

YLR164W	---	YLR164Wp is homologous to TIM18p	YNL300W	---	Hypothetical ORF
YPR015C	---	Hypothetical ORF	YGL101W	---	Hypothetical ORF
YHL035C	---	ABC transporter	YLR054C	---	Non-essential protein required for construction of the outer spore wall layers
YLR326W	---	Hypothetical ORF	YLR290C	---	Hypothetical ORF
YDL152W	---	---	YER185W	---	Hypothetical ORF
YIR036C	---	Hypothetical ORF	YNL141W	AAH1	adenine aminohydrolase (adenine deaminase)
YCL033C	---	Hypothetical ORF	YBR158W	AMN1	Involved in daughter cell separation and Chromosome STability
YLR077W	---	The authentic, non-tagged protein was localized to the mitochondria	YHR208W	BAT1	branched-chain amino acid transaminase
YBR070C*	---	Protein required for cell viability	YHR052W	CIC1	Core interacting component 1
YLR109W	AHP1	alkyl hydroperoxide reductase	YPL256C	CLN2	G1 cyclin
YJL115W	ASF1	anti-silencing protein	YNL112W	DBP2	ATP dependent RNA helicase|dead box protein
YML102W	CAC2	chromatin assembly factor-I (CAF-I) p60 subunit	YDL160C	DHH1	Cytoplasmic DExD/H-box helicase
YOR031W	CRS5	metallothionein-like protein	YJL157C	FAR1	Cdc28p kinase inhibitor
YDL101C*	DUN1	protein kinase	YIL131C	FKH1	forkhead protein
YDL018C	ERP3	p24 protein involved in membrane trafficking	YBR010W	HHT2	histone H3 (HHT1 and HHT2 code for identical proteins)
YIR038C	GTT1	glutathione transferase	YER110C	KAP123	karyopherin beta 4
YBR072W	HSP26	heat shock protein 26	YOL025W	LAG2	affects longevity
YIR037W	HYR1	glutathione-peroxidase (putative)	YDR234W	LYS4	homoaconitase
YOR328W	PDR10	ABC transporter (putative)|highly similar to Pdr5p	YIL106W	MOB1	Mps One Binder
YNL102W	POL1	DNA polymerase I alpha subunit p180	YDR033W	MRH1	Protein that localizes primarily to the plasma membrane
YER095W*	RAD51	Rad51p colocalizes to ~65 spots with Dmc1p prior to synapsis	YBR093C	PHO5	acid phosphatase
YNL312W	RFA2	29% identical to the human p34 subunit of RF-A|replication factor RF-A subunit 2	YGL028C	SCW11	glucanase
YJL026W*	RNR2	ribonucleotide reductase subunit	YGR245C	SDA1	Severe Depolymerization of Actin
YGL103W	RPL28	ribosomal protein L28 (L29) (rp44) (YL24)	YIL162W	SUC2	invertase (sucrose hydrolyzing enzyme)
YGR027C	RPS25A	ribosomal protein S25A (S31A) (rp45) (YS23)	YDR297W	SUR2	sphingosine hydroxylase
YER096W	SHC1	Sporulation-specific activator	YPR016C	TIF6	similar to human translation initiation factor 6 (eIF6);
			YDR120C	TRM1	N2,N2-dimethylguanosine-specific tRNA methyltransferase
			YBL004W	UTP20	U3 snoRNP protein
			YBR104W	YMC2	Putative mitochondrial inner membrane transporter

^a ^WT *S. cerevisiae *was exposed to enediynes or γ-radiation and regression analysis identified genes with increased transcriptional activation associated either with single-strand (left) or double-strand damage (right). Members of the DNA damage signature are highlighted in grey and denoted with an asterisk (*).
