# Supplementary material for: The DNA-damage signature in Saccharomyces cerevisiae is associated with single-strand breaks in DNA
Source: BMC Genomics. 2006 Dec 12;7:313. doi: 10.1186/1471-2164-7-313 (PMC1764021; doi:10.1186/1471-2164-7-313)
Supplement: Additional File 1 — Differential enrichment of GO categories identified in the comparison of yeast responses to enediynes and γ-radiation. The table lists the gene ontology categories identified as significantly enriched within the four sectors identified in the comparison of the enediynes response to that of γ-radiation. [file 1471-2164-7-313-S1.pdf]

R. Fry *et al*. Supplemental Table 1

Sector 1

Category (Gene Ontology)	p-value	Genes In Category from Cluster	k	f	sector
response to stress [GO:0006950]	3.72E-05	HSP26 YCL033C DUN1 RAD51 PRE3 ASF1 MSN4 AHP1 MSN2 CRS5	10	199	I
DNA replication, priming [GO:0006269]	3.79E-05	RFA1 POL1 RFA2	3	7	I
double-strand break repair [GO:0006302]	0.0002503	RFA1 RAD51 POL1 RFA2	4	30	I
DNA recombination [GO:0006310]	0.001153	RFA1 RAD51 MSC1 POL1 RFA2	5	76	I
response to biotic stimulus [GO:0009607]	0.002572	YCL033C DUN1 RAD51 ASF1 AHP1	5	91	I
DNA replication [GO:0006260]	0.002572	RFA1 RNR3 RNR2 POL1 RFA2	5	91	I
S phase of mitotic cell cycle [GO:0000084]	0.002828	RFA1 RNR3 RNR2 POL1 RFA2	5	93	I
DNA strand elongation [GO:0006271]	0.003028	RFA1 POL1 RFA2	3	28	I
double-strand break repair via synthesis-dependent strand annealing [GO:0045003]	0.004655	RAD51 POL1	2	10	I
post-replication repair [GO:0006301]	0.004655	RFA1 RFA2	2	10	I
DNA unwinding [GO:0006268]	0.005651	RFA1 RFA2	2	11	I
double-strand break repair via single-strand annealing [GO:0045002]	0.006736	RAD51 POL1	2	12	I
response to DNA damage [GO:0006974]	0.008349	DUN1 RAD51 ASF1	3	40	I

Sector 3

Category (Gene Ontology)	p-value	Genes In Category from Cluster	k	f	
chromatin assembly/disassembly [GO:0006333]	2.00E-06	HTA2 HHT1 HTB1 HTA1 HHF2	5	23	III
lysine biosynthesis [GO:0009085]	6.73E-05	LYS21 LYS4 LYS12	3	9	III
lysine metabolism [GO:0006553]	6.73E-05	LYS21 LYS4 LYS12	3	9	III
cell growth and/or maintenance [GO:0008151]	0.0003162	HTA2 HHT1 CHS2 PHO3 PHO5 YMC2 DUT1 CTP1 CHA1 LYS21 FAP7 RPL41A RRP1 TRM1	48	3657	III
aspartate family amino acid biosynthesis [GO:0009067]	0.0004276	LYS21 LYS4 LYS12	3	16	III
amino acid biosynthesis [GO:0008652]	0.001524	LYS21 LYS4 TRP2 BAT1 LYS12	5	89	III
amine biosynthesis [GO:0009309]	0.002036	LYS21 LYS4 TRP2 BAT1 LYS12	5	95	III
amino acid metabolism [GO:0006520]	0.002074	CHA1 LYS21 LYS4 TRP2 BAT1 LYS12	6	140	III
DNA packaging [GO:0006323]	0.00215	HTA2 HHT1 HTB1 HTA1 FKH1 HHF2	6	141	III
establishment and/or maintenance of chromatin architecture [GO:0006325]	0.00215	HTA2 HHT1 HTB1 HTA1 FKH1 HHF2	6	141	III
lysine biosynthesis, aminoadipic pathway [GO:0019878]	0.002432	LYS21 LYS4	2	8	III
amino acid and derivative metabolism [GO:0006519]	0.002839	CHA1 LYS21 LYS4 TRP2 BAT1 LYS12	6	149	III
amine metabolism [GO:0009308]	0.003035	CHA1 LYS21 LYS4 TRP2 BAT1 LYS12	6	151	III
lariat formation, 5'-splice site cleavage [GO:0006372]	0.003108	PRP28 PRP43	2	9	III
chromosome organization and biogenesis (sensu Eukarya) [GO:0007001]	0.004428	HTA2 HHT1 HTB1 HTA1 FKH1 HHF2	6	163	III
ribosomal large subunit biogenesis [GO:0042273]	0.00657	NSA2 TIF6	2	13	III
aspartate family amino acid metabolism [GO:0009066]	0.006869	LYS21 LYS4 LYS12	3	41	III
biosynthesis [GO:0009058]	0.007829	LYS21 RPL41A LYS4 GPI17 RPL27B PMI40 TRP2 TSC11 RPL22B RPL28 BAT1 LYS12 RPI1	14	731	III
nucleotide catabolism [GO:0009166]	0.009574	DUT1	1	1	III
pyrimidine nucleotide catabolism [GO:0006244]	0.009574	DUT1	1	1	III
deoxyribonucleoside triphosphate catabolism [GO:0009204]	0.009574	DUT1	1	1	III
nucleoside triphosphate catabolism [GO:0009143]	0.009574	DUT1	1	1	III
pyrimidine nucleoside triphosphate catabolism [GO:0009149]	0.009574	DUT1	1	1	III
cytosine transport [GO:0015856]	0.009574	FCY2	1	1	III
purine transport [GO:0006863]	0.009574	FCY2	1	1	III
unfolded protein response, target gene transcriptional activation [GO:0006990]	0.009574	HAC1	1	1	III
deoxyribonucleotide catabolism [GO:0009264]	0.009574	DUT1	1	1	III
pyrimidine deoxyribonucleotide catabolism [GO:0009223]	0.009574	DUT1	1	1	III

pyrimidine deoxyribonucleoside triphosphate catabolism [GO:0009213]	0.009574	DUT1	1	1	III
citrate transport [GO:0015746]	0.009574	CTP1	1	1	III
mitochondrial citrate transport [GO:0006843]	0.009574	CTP1	1	1	III

Sector 2

Category (Gene Ontology)	p-value	Genes In Category from Cluster	k	f	
mutagenesis [GO:0006280]	0.001277	POL30 CDC8	2	7	II
post-replication repair [GO:0006301]	0.002695	POL30 RFA3	2	10	II
DNA replication [GO:0006260]	0.005757	POL30 RNR4 RFA3 CDC8	4	91	II
S phase of mitotic cell cycle [GO:0000084]	0.006216	POL30 RNR4 RFA3 CDC8	4	93	II
dTTP metabolism [GO:0046075]	0.007978	CDC8	1	1	II
pyrimidine deoxyribonucleoside triphosphate biosynthesis [GO:0009212]	0.007978	CDC8	1	1	II
deoxyribonucleoside triphosphate biosynthesis [GO:0009202]	0.007978	CDC8	1	1	II
pyrimidine nucleoside triphosphate biosynthesis [GO:0009148]	0.007978	CDC8	1	1	II
dTDP biosynthesis [GO:0006233]	0.007978	CDC8	1	1	II
dTDP metabolism [GO:0046072]	0.007978	CDC8	1	1	II
pyrimidine deoxyribonucleoside diphosphate metabolism [GO:0009196]	0.007978	CDC8	1	1	II
deoxyribonucleoside diphosphate metabolism [GO:0009186]	0.007978	CDC8	1	1	II
nucleoside diphosphate metabolism [GO:0009132]	0.007978	CDC8	1	1	II
pyrimidine nucleoside diphosphate metabolism [GO:0009138]	0.007978	CDC8	1	1	II
pyrimidine deoxyribonucleoside diphosphate biosynthesis [GO:0009197]	0.007978	CDC8	1	1	II
deoxyribonucleoside diphosphate biosynthesis [GO:0009189]	0.007978	CDC8	1	1	II
nucleoside diphosphate biosynthesis [GO:0009133]	0.007978	CDC8	1	1	II
pyrimidine nucleoside diphosphate biosynthesis [GO:0009139]	0.007978	CDC8	1	1	II
dTTP biosynthesis [GO:0006235]	0.007978	CDC8	1	1	II

Sector 4

Category	p-value	Genes In Category from Cluster	k	f	
chromatin assembly/disassembly [GO:0006333]	0.0007564	HTB2 HHT1 HHT2	3	23	IV
response to osmotic stress [GO:0006970]	0.005358	MSN1 LAS17 IQG1	3	45	IV
response to abiotic stimulus [GO:0009628]	0.005677	STE6 CDC42 MSN1 LAS17 IQG1	5	145	IV
regulation of CDK activity [GO:0000079]	0.00613	CLB1 CLN2	2	15	IV
budding [GO:0007114]	0.00747	BUD4 CDC42 MUB1 LAS17	4	98	IV
sucrose catabolism [GO:0005987]	0.007978	SUC2	1	1	IV
sucrose metabolism [GO:0005985]	0.007978	SUC2	1	1	IV
nucleobase catabolism [GO:0046113]	0.007978	AAH1	1	1	IV
arginine catabolism to ornithine [GO:0019547]	0.007978	CAR1	1	1	IV
purine base catabolism [GO:0006145]	0.007978	AAH1	1	1	IV
meiotic G2/MI transition [GO:0008315]	0.007978	CLB1	1	1	IV
regulation of budding [GO:0007116]	0.007978	MUB1	1	1	IV
a-factor export [GO:0007325]	0.007978	STE6	1	1	IV
adenine catabolism [GO:0006146]	0.007978	AAH1	1	1	IV
chromosome organization and biogenesis (sensu Eukarya) [GO:0007001]	0.009227	HTB2 HEK2 HHT1 HHT2 HTZ1	5	163	IV
response to external stimulus [GO:0009605]	0.009613	HEK2 STE6 CDC42 MSN1 LAS17 IQG1	6	231	IV
