# Supplementary material for: The DNA-damage signature in Saccharomyces cerevisiae is associated with single-strand breaks in DNA
Source: BMC Genomics. 2006 Dec 12;7:313. doi: 10.1186/1471-2164-7-313 (PMC1764021; doi:10.1186/1471-2164-7-313)
Supplement: Additional File 2 — GO categories identified as associated with single-strand or double-strand damage. The table is a list of the gene ontology categories enriched in the gene sets that were identified as associated with single-strand or double-strand damage. [file 1471-2164-7-313-S2.pdf]

R. Fry *et al*. Supplemental Table 2

A, GO Biological Process (Single-strand break association)

Category	p-value	In Category from Cluster	k	f
response to stress [GO:0006950]	8.58E-07	HSP26 YCL033C DUN1 RAD51 HYR1 ASF1 AHP1 CRS5	8	199
response to biotic stimulus [GO:0009607]	1.45E-06	YCL033C DUN1 RAD51 HYR1 ASF1 AHP1	6	91
response to external stimulus [GO:0009605]	2.65E-06	YCL033C DUN1 RAD51 HYR1 ASF1 AHP1 CRS5 PDR10	8	231
cell communication [GO:0007154]	5.39E-05	YCL033C DUN1 RAD51 HYR1 ASF1 AHP1 CRS5 PDR10	8	348
double-strand break repair [GO:0006302]	0.000239	RAD51 POL1 RFA2	3	30
response to oxidative stress [GO:0006979]	0.0002904	YCL033C HYR1 AHP1	3	32
DNA replication, priming [GO:0006269]	0.0003432	POL1 RFA2	2	7
oxygen and reactive oxygen species metabolism [GO:0006800]	0.00038	YCL033C HYR1 AHP1	3	35
response to DNA damage [GO:0006974]	0.0005658	DUN1 RAD51 ASF1	3	40
double-strand break repair via synthesis-dependent strand annealing [GO:0045003]	0.0007298	RAD51 POL1	2	10
double-strand break repair via single-strand annealing [GO:0045002]	0.001065	RAD51 POL1	2	12
DNA repair [GO:0006281]	0.001216	RAD51 CAC2 POL1 RFA2	4	116
recombinational repair [GO:0000725]	0.001917	RAD51 POL1	2	16
double-strand break repair via homologous recombination [GO:0000724]	0.001917	RAD51 POL1	2	16
non-recombinational repair [GO:0000726]	0.003004	RAD51 POL1	2	20
DNA recombination [GO:0006310]	0.003646	RAD51 POL1 RFA2	3	76
induction of programmed cell death [GO:0012502]	0.004149	ASF1	1	1
induction of apoptosis [GO:0006917]	0.004149	ASF1	1	1
induction of apoptosis by intracellular signals [GO:0008629]	0.004149	ASF1	1	1
induction of apoptosis by DNA damage [GO:0008630]	0.004149	ASF1	1	1
strand invasion [GO:0042148]	0.004149	RAD51	1	1
mitotic recombination [GO:0006312]	0.004683	RAD51 POL1	2	25
DNA strand elongation [GO:0006271]	0.005855	POL1 RFA2	2	28
DNA replication [GO:0006260]	0.006047	RNR2 POL1 RFA2	3	91
S phase of mitotic cell cycle [GO:0000084]	0.006424	RNR2 POL1 RFA2	3	93
meiotic joint molecule formation [GO:0000709]	0.008281	RAD51	1	2
response to chemical substance [GO:0042221]	0.009563	CRS5 PDR10	2	36

B, GO Biological Process (Double-strand break association)

Category	p-value	In Category from Cluster	k	f
regulation of cell cycle [GO:0000074]	0.00128	SDA1 FKH1 FAR1 CLN2	4	105
purine base catabolism [GO:0006145]	0.004627	AAH1	1	1
nucleobase catabolism [GO:0046113]	0.004627	AAH1	1	1
sucrose metabolism [GO:0005985]	0.004627	SUC2	1	1
sucrose catabolism [GO:0005987]	0.004627	SUC2	1	1
adenine catabolism [GO:0006146]	0.004627	AAH1	1	1
start control point of mitotic cell cycle [GO:0007089]	0.009234	SDA1	1	2
adenine metabolism [GO:0046083]	0.009234	AAH1	1	2
