# Supplementary material for: The DNA-damage signature in Saccharomyces cerevisiae is associated with single-strand breaks in DNA
Source: BMC Genomics. 2006 Dec 12;7:313. doi: 10.1186/1471-2164-7-313 (PMC1764021; doi:10.1186/1471-2164-7-313)

Fry *et al.* Additional File 3: Cytotoxicity dose-response curves for *S. cerevisiae* strain DBY747 treated with enediynes

- Data represent mean of duplicate samples
- Data were fit to an equation for exponential decay
- 20-25% cytotoxicity window indicated with gray box
- Calculated values for neocarzinostatin:
  LD_{20} = 3.3 nM
  LD_{25} = 4.2 nM
- Calculated values for calicheamicin:
  LD_{20} = 1 nM
  LD_{25} = 1.4 nM

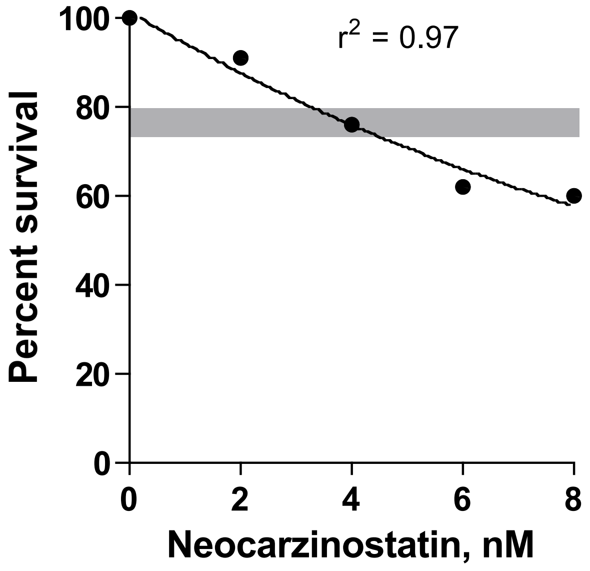

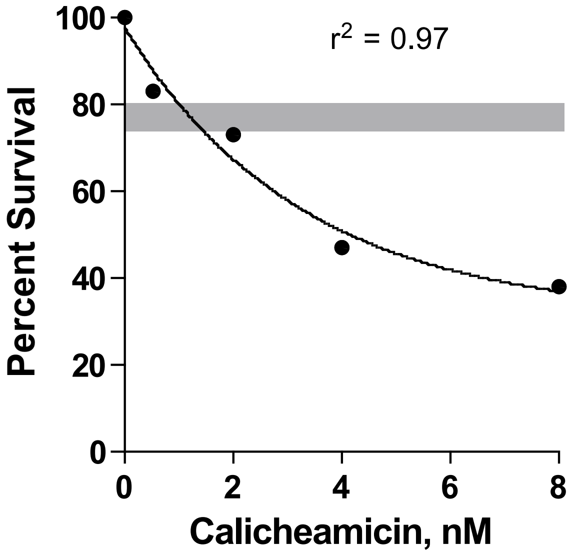

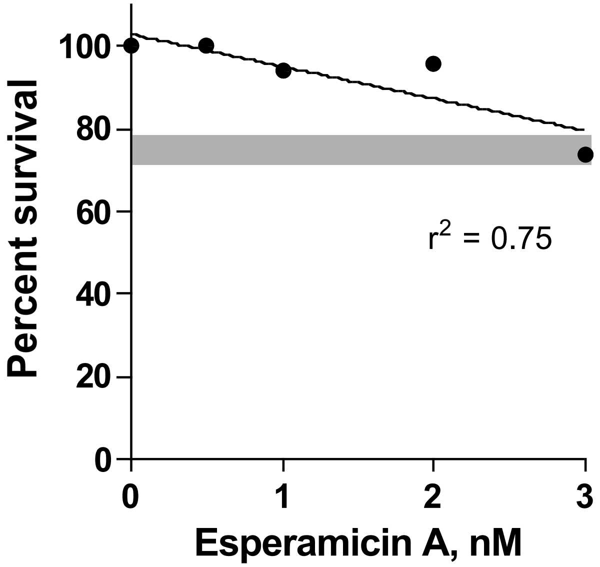

Supplement: Additional File 3 — Cytotoxicity dose-response curves for S. cerevisiae strain DBY747 treated with enediynes. The figure plots the cytotoxicity dose-response curve for exposure of strain DBY747 to enediynes. [file 1471-2164-7-313-S3.pdf]
